# Supplementary material for: Effect of 8-hydroxyquinoline and derivatives on human neuroblastoma SH-SY5Y cells under high glucose
Source: PeerJ. 2016 Aug 31;4:e2389. doi: 10.7717/peerj.2389 (PMC5012261; doi:10.7717/peerj.2389)

Fig.1 B

Cell viability (% of control)	8-Hydroxyquinoline (µM) – treated for 24 hr		
	**0**	**1**	**10**
**n1**	100	86.181	63.178
**n2**	100	116.604	74.519
**n3**	100	79.111	63.128
**n4**	100	92.181	73.850
**mean ± S.E.M**	100	93.52 ± 8.15	68.67 ± 3.19
P value		ns	< 0.01

Cell viability (% of control)	Clioquinol (µM) – treated for 24 hr		
	**0**	**1**	**10**
**n1**	100	114.385	103.271
**n2**	100	93.719	128.591
**n3**	100	100.153	74.166
**n4**	100	95.062	94.893
**mean ± S.E.M**	100	100.83 ± 4.73	100.23 ± 11.26
P value		ns	ns

Cell viability (% of control)	Nitroxoline (µM) – treated for 24 hr		
	**0**	**1**	**10**
**n1**	100	73.368	41.216
**n2**	100	99.712	44.217
**n3**	100	80.606	36.681
**n4**	100	92.088	34.568
**mean ± S.E.M**	100	86.44 ± 5.87	39.17 ± 2.18
P value		ns	< 0.001

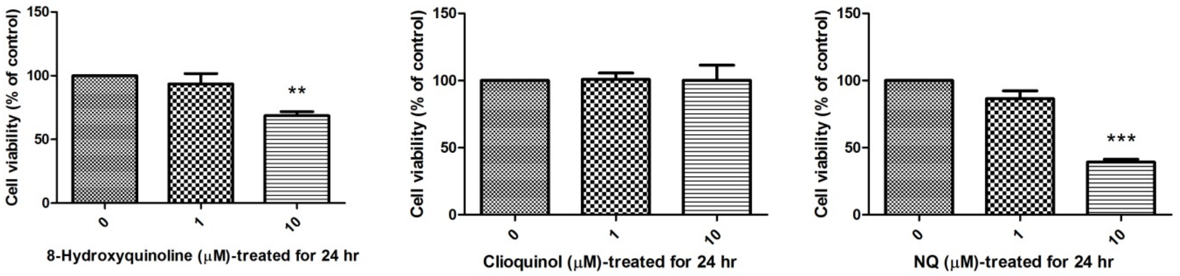

Supplement: Data S1 — B) Cells were treated with 8-hydroxyquinoline and derivative at 1 and 10 µM for 24 h. Cell viability was measured using the MTT assay and is presented as the percentage of control cells. The results are expressed as the mean ± S.E.M. of four independent experiments. One-way analysis of variance (ANOVA) and the Tukey-Kramer multiple comparisons test were performed for statistical analysis. **P < 0.01 and ***P < 0.001 compared with control. [file peerj-04-2389-s001.pdf]
