# Supplementary material for: Effect of 8-hydroxyquinoline and derivatives on human neuroblastoma SH-SY5Y cells under high glucose
Source: PeerJ. 2016 Aug 31;4:e2389. doi: 10.7717/peerj.2389 (PMC5012261; doi:10.7717/peerj.2389)

Fig.2 A

% cell viability	D-Glucose (mM), treated for 2 hr			
	**5.5**	**30**	**60**	**120**
**n1**	100	96.730	93.138	86.737
**n2**	100	96.644	91.017	87.392
**n3**	100	96.961	91.503	78.431
**n4**	100	95.204	89.586	77.798
**mean + S.E.M**	100	96.38 + 0.39	91.31 + 0.73	82.59 + 2.59
P value		ns	< 0.01	< 0.001

% cell viability	D-Mannitol (mM), treated for 2 hr			
	**5.5**	**30**	**60**	**120**
**n1**	100	101.607	97.444	94.290
**n2**	100	105.999	99.187	96.934
**n3**	100	87.610	101.328	101.327
**mean + S.E.M**	100	98.41 + 5.55	99.32 + 1.12	97.52 + 2.05
P value		ns	ns	ns

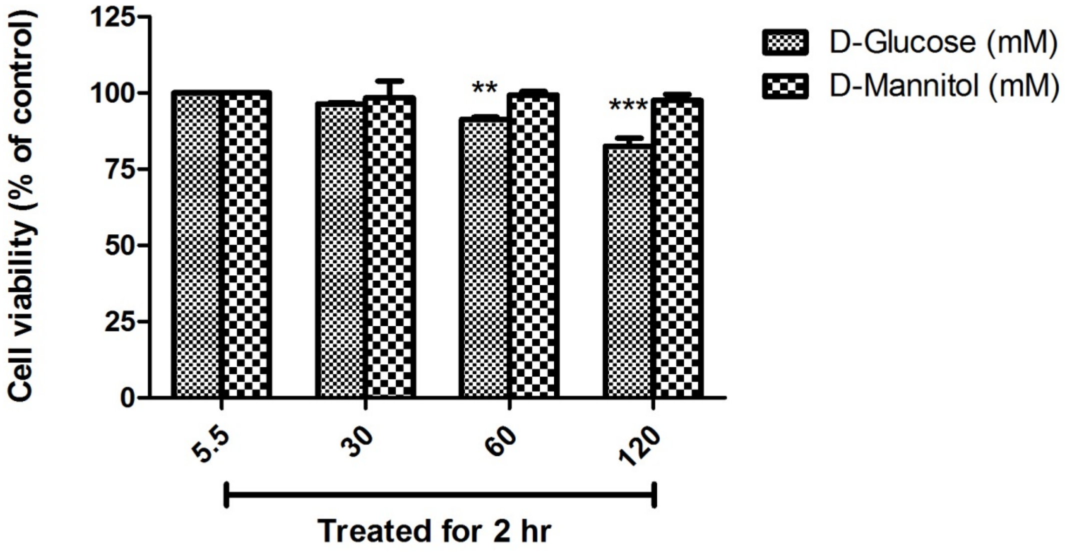

Supplement: Data S2 — Cells treated with D-glucose concentrations (30, 60 and 120 mM) for 2 h and 24 h were compared to cells treated with control medium containing 5.5 mM D-glucose and mannitol as an osmotic control. Cell viability was measured using the MTT assay. The results are expressed as the mean + S.E.M. of four independent experiments. One-way analysis of variance (ANOVA) and Tukey-Kramer multiple comparisons test were performed for statistical analysis, *P < 0.05, **P < 0.01 and ***P < 0.001 compared with the control at 2 h and ###P < 0.001 compared with control at 24 h. [file peerj-04-2389-s002.pdf]
