# Supplementary material for: Effect of 8-hydroxyquinoline and derivatives on human neuroblastoma SH-SY5Y cells under high glucose
Source: PeerJ. 2016 Aug 31;4:e2389. doi: 10.7717/peerj.2389 (PMC5012261; doi:10.7717/peerj.2389)

Fig.2 B

% cell viability	D-Glucose (mM), treated for 24 hr			
	**5.5**	**30**	**60**	**120**
**n1**	100	80.464	79.886	76.775
**n2**	100	93.309	80.626	78.790
**n3**	100	94.716	77.973	71.549
**n4**	100	87.897	75.436	68.760
**mean ± S.E.M**	100	89.10 ± 3.23	78.48 ± 1.16	73.97 ± 2.31
P value		< 0.05	< 0.001	< 0.001

% cell viability	D-Mannitol (mM), treated for 24 hr			
	**5.5**	**30**	**60**	**120**
**n1**	100	106.995	85.464	75.922
**n2**	100	98.479	77.328	73.115
**n3**	100	97.113	86.469	73.390
**n4**	100	87.697	86.616	79.310
**mean ± S.E.M**	100	97.57 ± 3.95	83.97 ± 2.23	75.43 ± 1.44
P value		ns	< 0.01	< 0.001

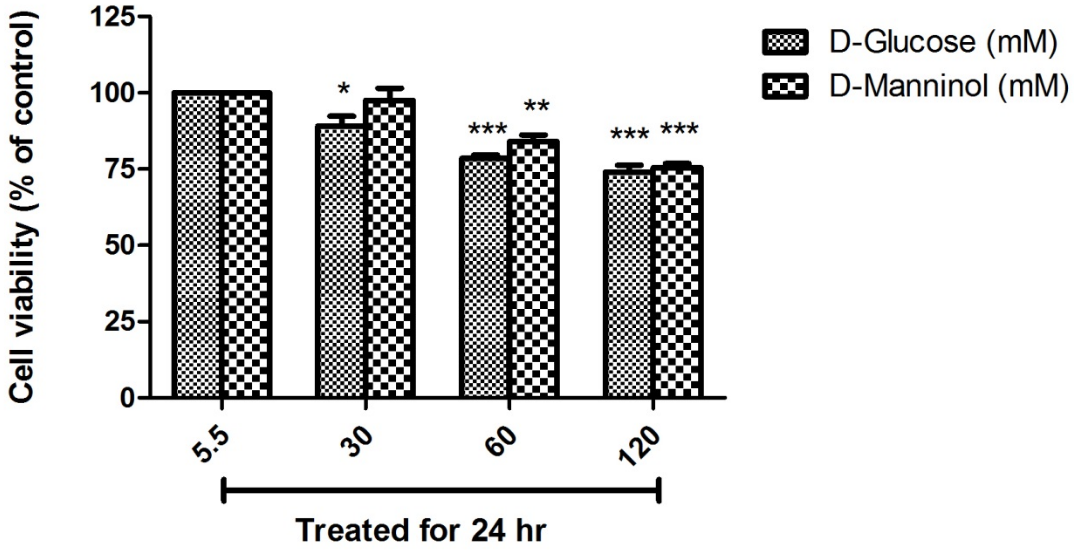

Supplement: Data S3 — Cells treated with D-glucose concentrations (30, 60 and 120 mM) for 2 h and 24 h were compared to cells treated with control medium containing 5.5 mM D-glucose and mannitol as an osmotic control. The results are expressed as the mean + S.E.M. of four independent experiments. One-way analysis of variance (ANOVA) and Tukey-Kramer multiple comparisons test were performed for statistical analysis, *P < 0.05, **P < 0.01 and ***P < 0.001 compared with the control at 2 h and ###P < 0.001 compared with control at 24 h. [file peerj-04-2389-s003.pdf]
