# Supplementary material for: Effect of 8-hydroxyquinoline and derivatives on human neuroblastoma SH-SY5Y cells under high glucose
Source: PeerJ. 2016 Aug 31;4:e2389. doi: 10.7717/peerj.2389 (PMC5012261; doi:10.7717/peerj.2389)

**Fig.2 C**

| Calpain expression | D-Glucose (mM), treated for 2 hr | | | |
|---|---|---|---|---|
| | **5.5** | **30** | **60** | **120** |
| **n1** | 100 | 92.985 | 115.593 | 139.111 |
| **n2** | 100 | 97.085 | 105.694 | 119.156 |
| **n3** | 100 | 111.922 | 122.141 | 147.935 |
| **n4** | 100 | 106.833 | 105.543 | 112.565 |
| **mean ± S.E.M** | 100 | 102.21 ± 4.35 | 112.24 ± 4.05 | 129.69 ± 8.30 |
| ***P value*** | | ns | ns | < 0.01 |

| Calpain expression | D-Glucose (mM), treated for 24 hr | | | |
|---|---|---|---|---|
| | **5.5** | **30** | **60** | **120** |
| **n1** | 100 | 98.722 | 106.388 | 140.877 |
| **n2** | 100 | 101.234 | 104.627 | 122.965 |
| **n3** | 100 | 108.888 | 118.575 | 135.733 |
| **n4** | 100 | 104.005 | 111.863 | 138.195 |
| **mean ± S.E.M** | 100 | 103.21 ± 2.18 | 110.36 ± 3.14 | 134.44 ± 3.97 |
| ***P value*** | | ns | ns | < 0.001 |

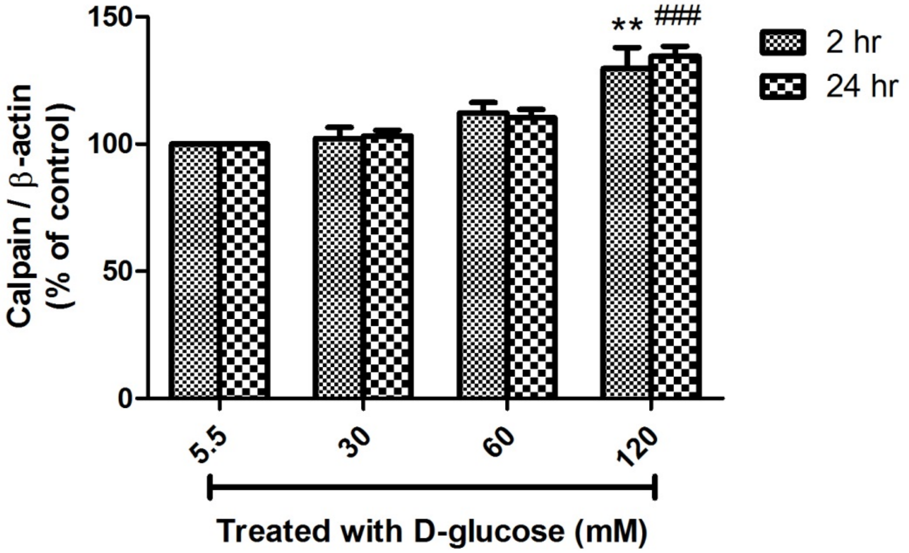

Supplement: Data S4 — D-glucose concentrations (30, 60 and 120 mM) for 2 h and 24 h were compared to cells treated with control medium containing 5.5 mM D-glucose. The levels of calpain was determined by Western blot analysis. Protein bands were quantified by densitometry, and their differences are represented in the graph as the ratio of calpain to β-actin. The results are expressed as the mean + S.E.M. of four independent experiments. One-way analysis of variance (ANOVA) and Tukey-Kramer multiple comparisons test were performed for statistical analysis, *P < 0.05, **P < 0.01 and ***P < 0.001 compared with the control at 2 h and ###P < 0.001 compared with control at 24 h. [file peerj-04-2389-s004.pdf]
