# Supplementary material for: Effect of 8-hydroxyquinoline and derivatives on human neuroblastoma SH-SY5Y cells under high glucose
Source: PeerJ. 2016 Aug 31;4:e2389. doi: 10.7717/peerj.2389 (PMC5012261; doi:10.7717/peerj.2389)

Fig.2 D

Calpastatin expression	D-Glucose (mM), treated for 2 hr			
	**5.5**	**30**	**60**	**120**
**n1**	100	103.875	119.323	114.443
**n2**	100	117.165	130.266	116.572
**n3**	100	97.265	129.268	104.628
**n4**	100	90.535	120.343	126.357
**mean ± S.E.M**	100	102.21 ± 5.68	124.80 ± 2.88	115.50 ± 4.46
P value		ns	< 0.01	ns

Calpastatin expression	D-Glucose (mM), treated for 24 hr			
	**5.5**	**30**	**60**	**120**
**n1**	100	93.33	85.257	73.252
**n2**	100	99.187	92.834	86.069
**n3**	100	99.716	89.654	76.005
**n4**	100	90.081	94.446	87.956
**mean ± S.E.M**	100	95.58 ± 2.34	90.55 ± 2.03	75.07 ± 4.35
P value		ns	ns	< 0.001

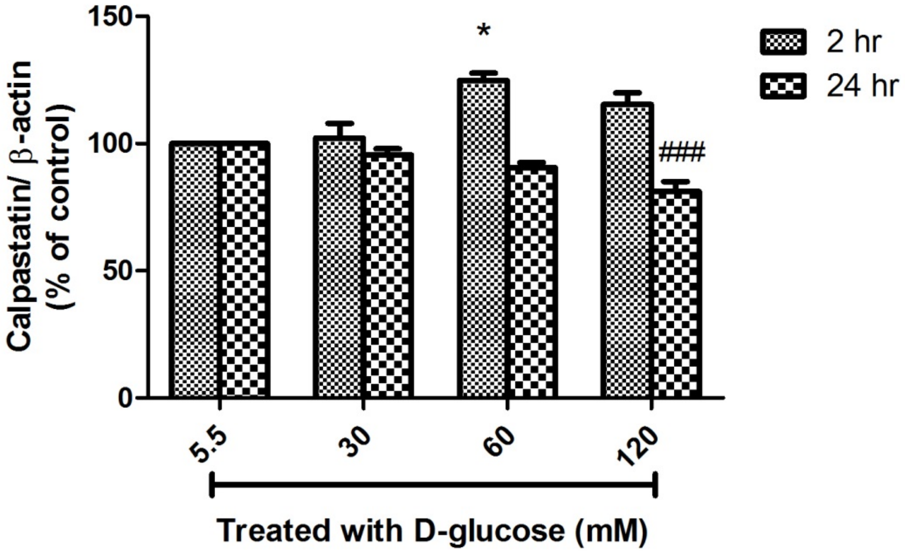

Supplement: Data S5 — Cells treated with D-glucose concentrations (30, 60 and 120 mM) for 2 h and 24 h were compared to cells treated with control medium containing 5.5 mM D-glucose. The levels of calpastatin was determined by Western blot analysis. Protein bands were quantified by densitometry, and their differences are represented in the graph as the ratio of calpastatin to β-actin. The results are expressed as the mean + S.E.M. of four independent experiments. One-way analysis of variance (ANOVA) and Tukey-Kramer multiple comparisons test were performed for statistical analysis, *P < 0.05, **P < 0.01 and ***P < 0.001 compared with the control at 2 h and ###P < 0.001 compared with control at 24 h. [file peerj-04-2389-s005.pdf]
