# Supplementary material for: Effect of 8-hydroxyquinoline and derivatives on human neuroblastoma SH-SY5Y cells under high glucose
Source: PeerJ. 2016 Aug 31;4:e2389. doi: 10.7717/peerj.2389 (PMC5012261; doi:10.7717/peerj.2389)

Fig.4 A

% cell viability	Control		8-Hydroxyquinoline		Clioquinol		Nitroxoline	
	5.5 mM Glucose	120 mM Glucose	5.5 mM Glucose	120 mM Glucose	5.5 mM Glucose	120 mM Glucose	5.5 mM Glucose	120 mM Glucose
**n1**	100	76.775	91.181	78.912	106.261	95.226	94.851	93.739
**n2**	100	78.790	100.951	93.723	103.843	92.314	106.08	95.001
**n3**	100	71.550	93.679	88.205	107.519	91.428	104.739	98.075
**n4**	100	68.760	91.349	86.726	99.374	94.438	102.218	96.079
**mean ± S.E.M**	100	73.97 ± 2.31	94.29 ± 2.29	86.89 ± 3.06	104.2 ± 1.80	93.35 ± 0.89	102.0 ± 2.51	95.72 ± 0.92
P value		< 0.01		< 0.01		< 0.001		< 0.001

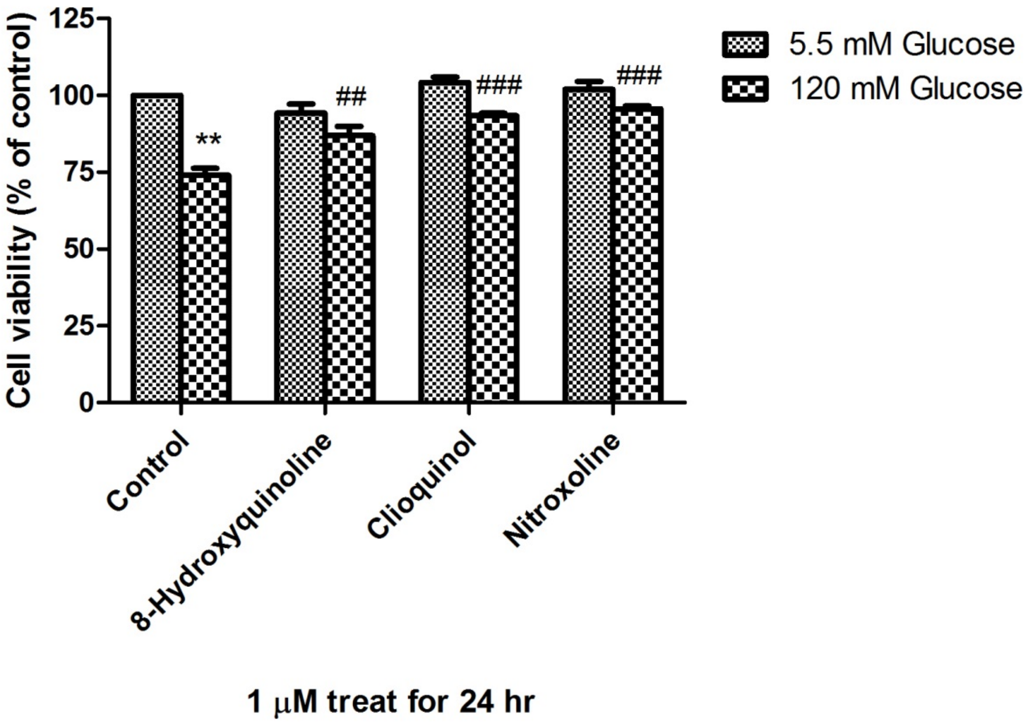

Supplement: Data S6 — Cells were treated with high glucose for 24 h. Some cells were pre-treated with 1 µM 8-hydroxyquinoline and derivatives for 2 h prior to incubation with 120 mM high glucose for another 24 h. The control cells were incubated with culture medium for 24 h. A, Cell viability was measured using the MTT assay. The results are expressed as the mean ± S.E.M. of four independent experiments. The results are expressed as the mean ± S.E.M. of three independent experiments. One-way analysis of variance (ANOVA) and the Tukey-Kramer multiple comparisons test were performed for statistical analysis. *P < 0.05, **P < 0.01 and ***P < 0.001 compared with the control and #P < 0.05, ##P < 0.01, ###P < 0.001 compared with high glucose-treated cells. [file peerj-04-2389-s006.pdf]
