# Supplementary material for: Effect of 8-hydroxyquinoline and derivatives on human neuroblastoma SH-SY5Y cells under high glucose
Source: PeerJ. 2016 Aug 31;4:e2389. doi: 10.7717/peerj.2389 (PMC5012261; doi:10.7717/peerj.2389)

Fig.4 B

	Calpain / β-actin (% of control)			
	Control	120 mM D-Glucose	1 μM 8-Hydroxyquinoline + 120 mM D-Glucose	1 μM 8-Hydroxyquinoline
**n1**	100	140.877	115.283	108.643
**n2**	100	122.965	99.251	96.181
**n3**	100	135.733	114.919	109.797
**mean ± S.E.M**	100	133.19 ± 5.32	109.82 ± 5.28	104.87 ± 4.359
P value		< 0.01	< 0.05	

Fig.4 C.

	Calpastatin / β-actin (% of control)			
	Control	120 mM D-Glucose	1 μM 8-Hydroxyquinoline + 120 mM D-Glucose	1 μM 8-Hydroxyquinoline
**n1**	100	87.956	85.845	81.265
**n2**	100	86.069	84.578	88.129
**n3**	100	75.076	99.970	99.542
**mean ± S.E.M**	100	83.034 ± 4.02	90.131 ± 4.93	89.645 ± 5.33
P value		< 0.05	ns	

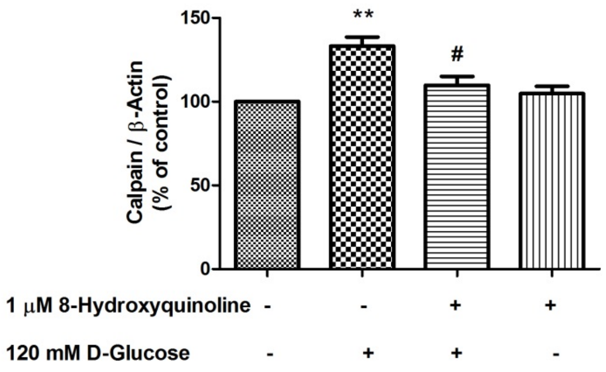

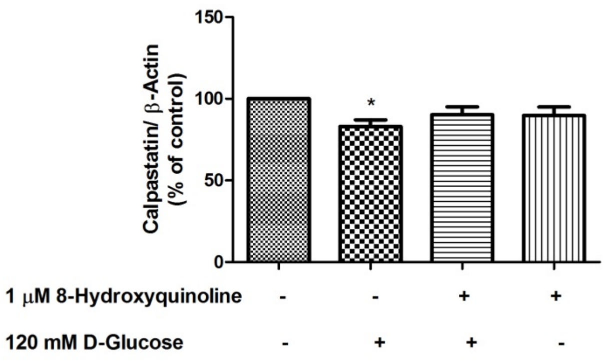

Supplement: Data S7 — Cells were treated with high glucose for 24 h. Some cells were pre-treated with 1 µM 8-hydroxyquinoline for 2 h prior to incubation with 120 mM high glucose for another 24 h. The control cells were incubated with culture medium for 24 h. Calpain and calpastatin expressions were determined by Western blot analysis. Protein bands were quantified by densitometry, and the changes are represented in the graph. Calpain and calpastatin expression are presented as the ratios of calpain or calpastatin/β-actin protein bands. The results are expressed as the mean ± S.E.M. of three independent experiments. One-way analysis of variance (ANOVA) and the Tukey-Kramer multiple comparisons test were performed for statistical analysis. *P < 0.05, **P < 0.01 and ***P < 0.001 compared with the control and #P < 0.05, ##P < 0.01, ###P < 0.001 compared with high glucose-treated cells. [file peerj-04-2389-s007.pdf]
