# Supplementary material for: Effect of 8-hydroxyquinoline and derivatives on human neuroblastoma SH-SY5Y cells under high glucose
Source: PeerJ. 2016 Aug 31;4:e2389. doi: 10.7717/peerj.2389 (PMC5012261; doi:10.7717/peerj.2389)

Fig.4 F

	Calpain / β-actin (% of control)			
	Control	120 mM D-Glucose	1 μM Nitroxoline + 120 mM D-Glucose	1 μM Nitroxoline
**n1**	100	140.877	106.273	109.495
**n2**	100	122.965	102.575	98.339
**n3**	100	135.733	107.552	109.959
**mean ± S.E.M**	100	133.19 ± 5.32	105.47 ± 1.49	105.93 ± 3.80
P value		< 0.001	< 0.01	ns

Fig.4 G.

	Calpastatin / β-actin (% of control)			
	Control	120 mM D-Glucose	1 μM Nitroxoline + 120 mM D-Glucose	1 μM Nitroxoline
**n1**	100	87.956	92.201	87.113
**n2**	100	86.069	88.606	96.521
**n3**	100	75.076	88.017	99.345
**mean ± S.E.M**	100	83.034 ± 4.02	89.61 ± 1.31	94.33 ± 3.70
P value		< 0.05	ns	ns

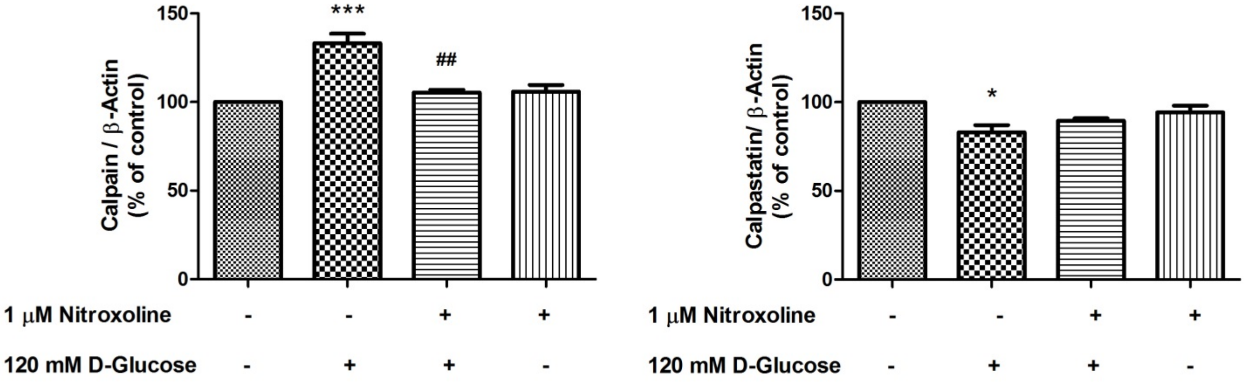

Supplement: Data S9 — Cells were treated with high glucose for 24 h. Some cells were pre-treated with 1 µM nitroxoline for 2 h prior to incubation with 120 mM high glucose for another 24 h. The control cells were incubated with culture medium for 24 h. Calpain and calpastatin expressions were determined by Western blot analysis. Protein bands were quantified by densitometry, and the changes are represented in the graph. Calpain and calpastatin expression are presented as the ratios of calpain or calpastatin/β-actin protein bands. The results are expressed as the mean ± S.E.M. of three independent experiments. One-way analysis of variance (ANOVA) and the Tukey-Kramer multiple comparisons test were performed for statistical analysis. *P < 0.05, **P < 0.01 and ***P < 0.001 compared with the control and #P < 0.05, ##P < 0.01, ###P < 0.001 compared with high glucose-treated cells. [file peerj-04-2389-s009.pdf]
